# Patterns of analgesic use, pain and self-efficacy: a cross-sectional study of patients attending a hospital rheumatology clinic

**DOI:** 10.1186/1471-2474-10-137

**Published:** 2009-11-10

**Authors:** Ruth Blamey, Kate Jolly, Sheila Greenfield, Paresh Jobanputra

**Affiliations:** 1 School of Health and Population Sciences, University of Birmingham, Edgbaston, Birmingham, B15 2TT, UK; 2 Department of Rheumatology, Selly Oak Hospital, University Hospital Birmingham NHS Foundation Trust, Raddlebarn Road, Birmingham, B29 6JD, UK; 3 Division of Infection & Immunity, University of Birmingham, Edgbaston, Birmingham, B15 2TT, UK

## Abstract

**Background:**

Many people attending rheumatology clinics use analgesics and non-steroidal anti-inflammatories for persistent musculoskeletal pain. Guidelines for pain management recommend regular and pre-emptive use of analgesics to reduce the impact of pain. Clinical experience indicates that analgesics are often not used in this way. Studies exploring use of analgesics in arthritis have historically measured adherence to such medication. Here we examine patterns of analgesic use and their relationships to pain, self-efficacy and demographic factors.

**Methods:**

Consecutive patients were approached in a hospital rheumatology out-patient clinic. Pattern of analgesic use was assessed by response to statements such as 'I always take my tablets every day.' Pain and self-efficacy (SE) were measured using the Western Ontario and McMaster Universities Osteoarthritis Index (WOMAC) and Arthritis Self-Efficacy Scale (ASES). Influence of factors on pain level and regularity of analgesic use were investigated using linear regression. Differences in pain between those agreeing and disagreeing with statements regarding analgesic use were assessed using t-tests.

**Results:**

218 patients (85% of attendees) completed the study. Six (2.8%) patients reported no current pain, 26 (12.3%) slight, 100 (47.4%) moderate, 62 (29.4%) severe and 17 (8.1%) extreme pain. In multiple linear regression self efficacy and regularity of analgesic use were significant (p < 0.01) with lower self efficacy and more regular use of analgesics associated with more pain.

Low SE was associated with greater pain: 40 (41.7%) people with low SE reported severe pain versus 22 (18.3%) people with high SE, p < 0.001. Patients in greater pain were significantly more likely to take analgesics regularly; 13 (77%) of those in extreme pain reported always taking their analgesics every day, versus 9 (35%) in slight pain. Many patients, including 46% of those in severe pain, adjusted analgesic use to current pain level. In simple linear regression, pain was the only variable significantly associated with regularity of analgesic use: higher levels of pain corresponded to more regular analgesic use (p = 0.003).

**Conclusion:**

Our study confirms that there is a strong inverse relationship between self-efficacy and pain severity. Analgesics are often used irregularly by people with arthritis, including some reporting severe pain.

## Background

Arthritis is a highly prevalent condition and can lead to physical impairments and psychological morbidity, including depression, anxiety and feelings of helplessness [1]. A long term need for analgesics and non-steroidal anti-inflammatory drugs (NSAIDs) is common, with many people also using non-pharmacological measures and complementary therapies [2,3]. However, previous research suggests that patients with arthritis commonly do not take NSAIDs as prescribed; taking pain medication in lower doses and less frequently than recommended [4]. Analgesics may be more effective for people with chronic pain when taken regularly and pre-emptively, thus decreasing the peak-and-trough effect of intermittent dosing. This, at least theoretically, enables more continuous pain relief [5] and reduces the likelihood of movement related pain. Research into arthritis patients' use of pain killers has traditionally focused on adherence to NSAIDs [6]. Methods of measuring compliance have included recording proportion of medication taken [7], assaying drug levels [8], self-reported adherence [9-11] and recording non-adherent behaviours such as missing doses [7]. In chronic conditions such as arthritis analgesics may be prescribed to be taken 'as needed' especially where the severity of pain fluctuates, so we have chosen to investigate the pattern of patients' use of pain killers rather than their actual adherence to prescribed regimens. In this study 'patterns' refers to the way in which people take analgesics on a daily basis.

Self-efficacy is defined as an individual's judgement of their capability to carry out a course of action required to achieve a desired goal [12] and in this study refers to a patient's perception of their ability to manage arthritis and its symptoms. Higher self-efficacy scores have been associated with better adherence to non-analgesic medication [13,14], lower pain intensity [15,16] and less functional impairment [17-19]. The relationship between self efficacy, pain and patterns of analgesic use is unclear. The aims of this study were to explore patterns of analgesic use among patients attending rheumatology clinics and the relationship between analgesic use, self-efficacy, pain and demographic characteristics.

## Methods

### Setting & Population

Our study was conducted in the outpatient rheumatology clinics of Selly Oak Hospital, Birmingham, which were provided by 11 clinicians. The majority of patients had some form of arthritis; a minority had some form of soft tissue musculoskeletal pain. Patients attending rheumatology clinics suffer mostly from an inflammatory arthritis and commonly endure skeletal pains from a variety of causes, including secondary osteoarthritis and myofacial pains [20]. We chose not to seek any further diagnostic information as we were interested in this heterogeneous population as a whole and the way these individuals responded to musculoskeletal pain. The term arthritis will be used in this article to describe the problems suffered by our study population.

Consecutive patients with any form of arthritis or pain of least 3 months duration and who were able to speak English were approached. Eligible patients were given an information leaflet and those giving written consent were asked to complete a questionnaire. Data were collected over 4 weeks during February and March 2007. Patients with a shorter history of arthritis were excluded because we wished to study established patterns of analgesic use and we believed that 3 months was a reasonable period for these to develop. Questionnaires were self-completed by 81.2% of patients. One researcher (RB) recorded the responses of those unable to write either due to arthritis affecting their hands or ability to speak but not write English.

**Figure 1:**
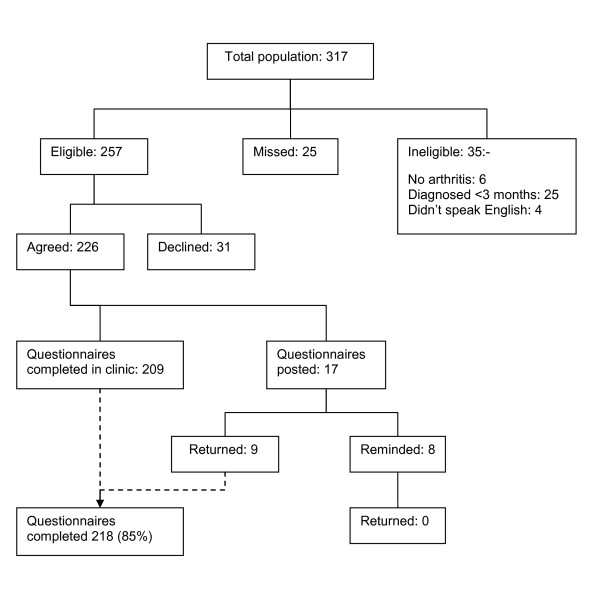
**Patient recruitment**.

Patients unable to complete questionnaires during their visit to the clinic were given a reply paid pre-addressed envelope and asked to return it by mail. Those who had not returned their questionnaire within two weeks were sent a reminder and a new questionnaire by mail (Figure 1).

### Questionnaires

Demographic data including gender, age, ethnicity (classified according to the 2001 census) [21] and duration of arthritis were collected. The shorter 8-item version of the Arthritis Self-Efficacy Scale (ASES) was used to assess self-efficacy for managing arthritis [22]. The ASES has been shown to have acceptable construct and concurrent validity and test-retest reliability [22]. The short-form version has been demonstrated to have good psychometric characteristics (high internal consistency [Cronbach's alpha = 0.89] and minimal floor and ceiling effects) [17]. In order to make this questionnaire more understandable for British patients the word 'blue' in the phrase 'if you are feeling blue' was replaced by the word 'low'. Each question of the ASES self-efficacy scale was answered on a scale of 1 to 10 (1 being very uncertain - low self-efficacy; 10 being very certain - high self-efficacy). The mean of the responses gave the self-efficacy rating. Pain was assessed by using the Western Ontario and McMaster Universities Osteoarthritis Index (WOMAC) pain sub-scale. Each of 5 questions is answered on a five point Likert scale (0 = no pain to 4 = extreme pain), the summation of which gives the participant's pain score. The WOMAC scale is widely used and its validity and reliability have been established in rheumatoid arthritis (RA) and osteoarthritis (OA) [23,24].

A series of statements was used to assess patients' pattern of analgesic use. (Table 1) In this study the term 'analgesics' included paracetamol (acetaminophen), compound analgesics (including paracetamol and codeine) and NSAIDs. Standard measures of adherence, such as proportion of medication taken, were not used in this study for several reasons. Firstly it was our intention to investigate how patients are using their analgesics, not simply if they were taking a certain amount. Secondly analgesics are often not prescribed to be taken a certain number of times a day but 'as needed'. Also, patients in pain may not be receiving prescription analgesics or NSAIDs but may be taking over the counter preparations.

**Table 1:** Pattern of analgesic use according to severity of pain

**Statement**	Number (%) of patients agreeing* with statement in each pain category (score range)				
	
	**None (n = 6)** **(0 to <0.5)**	**Slight (n = 26)** **(0.5 to <1.5)**	**Moderate (n = 100)** **(1.5 to <2.5)**	**Severe (n = 62)** **(2.5 to <3.5)**	**Extreme (n = 17)** **(3.5 to 4)**
1. I always take my tablets everyday	2 (33%)	9 (35%)	66 (65%)	47 (72%)	13 (77%)

2. I only take a tablet when the pain gets too bad	5 (83%)	14 (54%)	45 (45%)	23 (35%)	5 (29%)

3. I vary how I take the tablets depending on how bad the pain is	3 (50%)	14 (54%)	54 (54%)	30 (46%)	6 (35%)

4. When I'm having a bad patch I take my tablets everyday, otherwise I only take them when I feel I need them	3 (50%)	14 (54%)	44 (44%)	20 (31%)	3 (18%)

5. I usually take the tablets before I go to bed	1 (17%)	6 (23%)	37 (37%)	24 (37%)	9 (53%)

6. I usually take the tablets before exercise	1 (17%)	3 (12%)	34 (34%)	20 (31%)	4 (24%)

* Patients were given the following instruction: 'These questions are about how you take the PAIN KILLER medications the doctor is currently giving you for you arthritis. Please circle the number that describes how much you agree with the statement'.

Scores were: 1 = strongly disagree; 2 = moderately disagree; 3 = slightly disagree; 4 = slightly agree; 5 = moderately agree; 6 = strongly agree.

Statement Number (percentage) of participants who answered the question

1. 197 (90)

2. 193 (89)

3. 189 (87)

4. 193 (89)

5. 191 (88)

6. 184 (84)

It was our aim to investigate how patients managed their analgesics in relation to their pain and related factors such as exercise and sleep. A recent study exploring osteoarthritis patients' management of pain and analgesics found that many patients varied their use of pain killers and were often reluctant to take analgesics unless unable to tolerate the pain [4]. Accordingly, statements relating to regular use; use only in significant pain and varying use depending on level of pain were included in this study. Both osteoarthritis and rheumatoid arthritis can be characterised by flare ups and remissions of symptoms. A statement relating to changes in analgesic use during flare ups was therefore also included. Sale et al. [4] also found that many patients had been advised to take analgesics before activity; however their participants did not report taking their analgesics in this way. Pain can often cause sleeping problems for patients with arthritis. A study involving clients from the Observational Arthritis Study in Seniors [25] found that 81% of patients had problems with sleep maintenance. We therefore also included statements related to analgesic use before exercise and sleep. Descriptors were discussed with clinicians and piloted by eight patients before use; no changes were made as a result of the pilot.

Patients recorded, on a 6-point Likert scale, whether they agreed (strongly agree = 6) or disagreed (strongly disagree = 1) with each statement. For questions where the more strongly the patient agreed with the statement the less regularly they took their analgesics, e.g. 'I only take a tablet when the pain gets too bad,' the scores were reversed. This allowed high scores for all questions to relate to more regular analgesic use. The scores for the four questions related to regularity of use (1-4 table 1) were then summated to provide an overall measure of how regularly patients were using analgesics. The internal reliability of the 'analgesic regularity' questionnaire was good, with a Cronbach's alpha of 0.82. Inter-item correlations ranged from 0.35 to 0.62 and item-total correlations from 0.49 to 0.74.

### Ethical approval

Ethical approval was granted by the South Birmingham Research Ethics Committee.

### Statistical considerations and analyses

All data were entered into Microsoft Excel and statistical analyses were performed in SPSS v15 for Windows. The WOMAC pain scores were grouped into 5 categories based on the original categories used to answer the pain questions: no pain (0-0.499), slight pain (0.5-1.499), moderate pain (1.5-2.499), severe pain (2.5-3.499) and extreme pain (3.5-4). The pattern of analgesic use at each level of pain was examined. T-Tests were also used to examine the differences in mean pain score between those agreeing and disagreeing with statements regarding analgesic use. As this involved multiple comparisons a more conservative significance level (p < 0.01) was used for these analyses, otherwise p < 0.05 was used. To investigate the relative influence of factors on regularity of analgesic use, simple and multiple regression analyses were undertaken. Firstly, simple regression analyses were undertaken between the predictor variables. A significant relationship was found between age and duration of the disease, but both were retained in the model. Age, gender, ethnicity, duration of disease, severity of pain and self-efficacy were entered simultaneously into the model. Ethnicity was entered as a binary variable (white British or other ethnic group) due to the relatively small number of participants from a minority ethnic group. An analysis of the residuals identified that they were normally distributed, so the assumptions for the model were met. Similar methods were used to investigate the influence of age, gender, ethnicity, duration of disease, regularity of analgesic use and self-efficacy on reported pain.

## Results

### Patient characteristics

Two hundred and eighteen patients completed questionnaires, providing a response rate of 85% (figure 1). The mean age of participants was 55 years (SD 14.8), 155 (71%) were female and patients had had arthritis for a median of 10 years (IQR 5, 20). Six (2.8%) patients reported having no current pain, 26 (12.3%) described their pain as slight, 100 (47.4%) moderate, 62 (29.4%) severe and 17 (8.1%) extreme. A majority of patients were white (183, 84%), twenty one (10%) Asian or British Asian, seven (3%) were mixed race and seven (3%) had a Black, or Black British background.

### Use of analgesics

Use of paracetamol (acetaminophen) was reported by 65 (29.8%) of participants; compound analgesics (including paracetamol and codeine) by 111 (50.9%) and NSAIDs by 71 (32.6%).

### Pattern of analgesic use

The majority of patients in extreme pain reported always taking their analgesics everyday, falling to less than a third of those in no pain. A large proportion of those in no (current) pain and slight pain reported only taking analgesics when they felt their pain was too severe, a practice less common amongst those in greater pain; yet almost one third of patients in extreme pain still only used analgesics when their pain became 'too bad'. A large proportion of patients with all severities of pain varied how they took their analgesics depending on their level of pain. Patients in slight or no pain were much more likely to only take analgesics regularly during 'bad patches' than those in extreme pain. Details are given in table 1.

The proportion of patients reporting taking analgesics before bed increased with the level of pain suffered. There was no distinct pattern in those who took analgesics before exercise. Very few of those in slight or no pain took analgesics before exercise compared with approximately one third of those in moderate and severe pain and one quarter of patients in extreme pain.

Patients who reported always taking their analgesics daily had a higher mean pain score than those who did not (details shown in table 2). Those who reported only taking analgesics when the pain got too bad had a lower pain score compared with those who did not. There was no significant difference in pain severity between those who varied how they took their analgesics depending on their level of pain and those who did not. However, there was a trend towards those who varied their analgesics in this way having slightly lower pain scores. Patients who only took their analgesics regularly when they were having a bad patch reported less pain than those who did not. In terms of pre-emptive analgesic use, patients taking their tablets before bed reported higher pain scores than those who did not, but this was not significant. There was no significant difference in pain related to taking analgesics before exercise.

**Table 2:** T-Tests exploring differences in reported pain between those agreeing and disagreeing with pain management strategies

**Statement**	**Agree with statement**		**Disagree with statement**		
	**Mean pain score**	**Standard deviation**	**Mean pain score**	**Standard deviation**	**p-value**
1. I always take my tablets everyday	12.0	3.9	9.6	4.6	<0.001

2. I only take a tablet when the pain gets too bad	10.4	4.3	12.2	3.9	0.003

3. I vary how I take the tablets depending on how bad the pain is	10.9	4.0	11.5	4.7	0.343

4. When I'm having a bad patch I take my tablets everyday, otherwise I only take them when I feel I need them	10.2	4.0	12.0	4.3	0.006

5. I usually take the tablets before I go to bed	12.0	4.0	10.6	4.4	0.034

6. I usually take the tablets before exercise	11.7	3.7	10.9	4.6	0.238

### Predictors of regularity of analgesic use

In simple linear regression, pain was the only variable significantly associated with regularity of analgesic use: a greater pain score (i.e. more pain) corresponded to more regular analgesic use. With multiple linear regression only pain was significantly associated with regularity of analgesic use (Table 3).

**Table 3:** Factors affecting reported pain and regularity of analgesic use: Simple and multiple linear regression.

**Variable**	**Factors affecting regularity of analgesic use**						**Factors affecting pain severity**					
	***Simple Linear Regression***			Multiple Linear Regression*			***Simple Linear Regression***			***Multiple Linear Regression#***		

	**β** **(9%% CI)**	**SE**	**p value**	**β** **(95% CI)**	**SE**	**p value**	**β** **(95% CI)**	**SE**	**p value**	**β** **(95% CI)**	**SE**	**p** **value**

Pain	0.35 (0.12 to 0.58)	0.12	0.003	0.37 (0.11 to 0.63)	0.13	0.006	-	-	-	-	-	-

Self-efficacy	-0.15 (-0.67 to 0.38)	0.27	0.578	0.09 (-0.49 to 0.67)	0.29	0.768	-0.85 (-1.13 to -0.58)	0.14	<0.001	-0.82 (-1.13 to -0.52)	0.16	<0.001

Gender	0.83 (-1.40 to 3.06)	1.13	0.464	0.64 (-0.60 to 2.87)	1.13	0.574	0.58 (-0.68 to 1.84)	0.64	0.368	0.52 (-0.77 to 1.80)	0.65	0.431

Age	0.04 -0.03 to 0.1)	0.03	0.245	0.04 (-0.03 to 0.11)	0.04	0.308	0.01 (-0.03 to 0.05)	0.02	0.53	0.01 (-0.03 to 0.05)	0.02	0.541

Ethnicity	1.69 (-0.68 to 4.07)	1.20	0.161	0.98 (-1.54 to 3.5)	1.28	0.446	1.46 (0.12 to 2.81)	0.68	0.034	1.39 (0.04 to 2.82)	0.73	0.057

Disease duration	0.04 (-0.05 to 0.14)	0.05	0.4	0.03 (-0.08 to 0.13)	0.05	0.629	-0.01 (-0.07 to 0.05)	0.03	0.68	-0.01 (-0.07 to 0.05)	0.03	0.769

Regularity of analgesic use	-	-	-	-	-	-	0.14 (0.05 to 0.23)	0.05	0.003	0.12 (0.04 to 0.21)	0.04	0.006

*Percentage of explained variance 3.7

Variables adjusted for: pain, self-efficacy, gender, age, ethnicity, disease duration

# Percentage of explained variance 13.5

Variables adjusted for: self-efficacy, gender, age, ethnicity, disease duration, regularity of analgesic use

### Predictors of pain severity

The degree of pain reported was not associated with age, gender or duration of arthritis, but was significantly associated with self-efficacy, regularity of analgesic use and ethnicity. Greater self-efficacy corresponded to less pain. Patients from ethnic minorities had an average pain score of 12.4 compared to 10.9 (p = 0.034) for those of white British ethnicity in simple linear regression (Table 3). In multiple linear regression where age, gender, duration of arthritis, ethnicity, regularity of analgesic use and self-efficacy were entered simultaneously, lower self efficacy and regular use of analgesics remained significantly associated with more pain.

### Specific self-management techniques

Patients commonly used other methods of managing pain. Many used rest (180, 84%), exercise (116, 54%), heat (101, 47%) or a bath or a spa (83, 38%). Forty two (20%) were currently being treated by a physiotherapist and 40 (19%) participants reported using a form of complementary therapy.

## Discussion

In this study we have explored the way in which patients attending rheumatology clinics manage their analgesics, and the relationships between pain, self-efficacy and how regularly analgesics are used.

Previous studies indicate that patients with RA [26] and OA [4] may be taking analgesics less frequently than prescribed: a finding that was replicated in our study. We found, however, that people in more pain tended to take analgesics more regularly and pre-emptively (particularly before sleep). (The size of the difference in WOMAC pain scores between those agreeing and disagreeing with statements regarding regularity of use is similar to that obtained by analgesic medication in patients with arthritis [27]). However, patterns of pre-emptive analgesic use before exercise were more complex. Those with moderate pain more frequently took medication before exercise than those with slight pain, but those in severe pain were less likely to do so, perhaps because exercise capacity was limited in those with severe pain or our study may have been underpowered to detect a difference. By contrast a smaller but similar study of RA patients found no relationship between pain and regularity of analgesic use [28].

In our study, a majority of patients (18, 64%) agreed with the statement 'I always take my tablets everyday'. A large proportion of patients, however, varied their use of analgesics depending on their current level of pain; including over a third of people reporting severe pain, who only used their medication on demand, or at times when pain was unbearable. This approach is at odds with recommendations in guidelines for health professionals, in which pre-emptive and regular use of analgesics is recommended in order to prevent breakthrough and movement related pain [29]. Recent draft guidance from the National Institute for Health and Clinical Excellence recognised the surprising lack of data on how patients use analgesics in OA [28,30]. Also surprisingly, publications for patients by respected arthritis organisations provide no guidance on how analgesics should be used [31], although it is relevant that disease knowledge is poorly related to patients' beliefs about medication [32]. Education and advice about analgesic use is likely to be provided by clinicians during normal clinical contacts. The clinic from which these patients were drawn also ran education classes for patients with newly diagnosed inflammatory arthritis. However, it is likely that few of the patients in this study had recently attended such classes: the median duration of arthritis in our population was 10 years.

Analgesic medication appears to be seen differently to other medications in that purposeful dose restraint is common and often seen as acceptable [4]. Patients may have many reasons for limiting their intake of analgesics in this way. A dislike of tablet taking despite, paradoxically, relatively common use of complementary medications has been previously found and is supported by our study [4]. Fears about dependency have also been well reported [4] and, in our experience, patients often express concerns about developing tolerance to analgesics. Use of regular medication is perceived by many people to indicate a loss of autonomy and to imply weakness [33]. Limiting analgesic consumption may, like the widespread use of complementary therapies, reflect an affirmation of autonomy or personal empowerment [34]. Beliefs and concerns about medicines are reported to influence adherence to medication. Indeed, medication beliefs have been found to have a greater influence on adherence in chronic illness than clinical and sociodemographic factors [35].

A variety of psychosocial factors are known to influence pain severity, which in turn may influence patterns of analgesic use. We found, like others [36], that patients from ethnic minorities reported more pain. However, only 35 (16%) patients from ethnic minorities were included in our population. This also prevented us from exploring the differences in pattern of analgesic use by severity of pain in each ethnicity, as the number of patients in each category was very small.

A measure of self efficacy was included in our analysis. This is a task specific trait [15] which seeks to ascertain how capable an individual believes they are of carrying out a course of action or behaviour needed to achieve a goal [12]; here, the management of their pain. The concept of self-efficacy is widely accepted and acknowledged to be important in the study of pain. We found, like others [37], that patients with higher perceived self efficacy reported less pain. Cross-sectional measurements of self-efficacy are believed to be a meaningful indicator of daily pain and coping, with greater self-efficacy often leading to more effective pain coping mechanisms [28]. Indeed, people with high levels of self efficacy (for pain) have reported less pain then those with low self-efficacy when subjected to painful stimuli in a laboratory [37]. Self efficacy was not, however, associated with regularity of analgesic use reported in our study, after the influence of pain was taken into account. No other measures were included in our study and whilst many have overlapping features, other psychological measures may have provided more insight into the reasons behind patterns of analgesic use. The low variance explained by the multivariate models for severity of pain (R^2^13.5%) and for regularity of analgesic use (R^2^3.7%) highlight the fact that that other psychological factors may be more influential. Beliefs about pain and analgesics [38,39], coping mechanisms [28], depression [40,41], anxiety [41] and psychological stress [42] may all be of importance in understanding patients' experience of arthritis, pain and their analgesic use.

Our study was cross-sectional and therefore has important limitations particularly because we do not know whether the patterns of analgesic use and perceived self efficacy, remain stable and whether the correlations reported hold true over time. To achieve a high response rate and generalisability we focused on the factors relating to pain that we were most interested in, such as self-efficacy. However we acknowledge that the investigation of other factors such as depression is important and may be worth further study in the future. We did not assess the psychometric properties of the patterns of analgesic use scale prior to its use in this study. It was not our intention to develop a new scale and to report the properties of this scale. We chose our questions to gain an insight into how patients managed their analgesics on a daily basis. A majority of questionnaires were self-completed (81%) but in 19% of cases (41 participants) a researcher recorded the answers. The most common reason for this was difficulty with writing due to arthritis; only 3 of the 41 participants who were assisted were from an ethnic minority. Considering that 16% of the total study population were from ethnic minorities, problems in participants' ability to write English were not a significant factor in this study and are unlikely to result in any bias. Important strengths of our study were an excellent response rate, use of well validated measures, a heterogeneous population, inclusion of consecutive patients and availability of a researcher to help with clarification when patients were completing questionnaires.

## Conclusion

We found that self-efficacy, regularity of analgesic use and ethnicity influenced the degree of pain reported by patients. The majority of patients in this hospital outpatient sample (85%) reported moderate or more severe pain. People with greater pain reported more regular analgesic use but a large proportion of patients, including some in severe pain, varied their consumption of analgesics greatly. This may be because of patients' beliefs and concerns about medications. Health professionals who recognise such concerns during consultations may be better able to guide patients and encourage more optimal patterns of analgesic use and promote better function.

## Competing interests

The authors declare that they have no competing interests.

## Authors' contributions

RB was involved in the conception and design of the study, collected data, did statistical analyses and drafted the manuscript. KJ co-supervised the project, was involved in the conception and design of the study, assisted with data analysis and helped revise the manuscript. PJ co-supervised the research, was involved the conception of the research, and helped draft and revise the manuscript. SG was involved in the conception of the study and critically reviewing drafts of the manuscript. This research formed part of RB's undergraduate dissertation. All authors read and approved the final manuscript.

## Pre-publication history

The pre-publication history for this paper can be accessed here:


